# Integrating Voice Quality Cues in the Pitch Perception of Speech and Non-speech Utterances

**DOI:** 10.3389/fpsyg.2018.02147

**Published:** 2018-11-29

**Authors:** Jianjing Kuang, Mark Liberman

**Affiliations:** Department of Linguistics, University of Pennsylvania, Philadelphia, PA, United States

**Keywords:** pitch perception, voice quality, spectral cues, speech perception, cue integration, prosody, speech normalization

## Abstract

Pitch perception plays a crucial role in speech processing. Since F0 is highly ambiguous and variable in the speech signal, effective pitch-range perception is important in perceiving the intended linguistic pitch targets. This study argues that the effectiveness of pitch-range perception can be achieved by taking advantage of other signal-internal information that co-varies with F0, such as voice quality cues. This study provides direct perceptual evidence that voice quality cues as an indicator of pitch ranges can effectively affect the pitch-height perception. A series of forced-choice pitch classification experiments with four spectral conditions were conducted to investigate the degree to which manipulating spectral slope affects pitch-height perception. Both non-speech and speech stimuli were investigated. The results suggest that the pitch classification function is significantly shifted under different spectral conditions. Listeners are likely to perceive a higher pitch when the spectrum has higher high-frequency energy (i.e., tenser phonation). The direction of the shift is consistent with the correlation between voice quality and pitch range. Moreover, cue integration is affected by the speech mode, where listeners are more sensitive to relative difference within an utterance when hearing speech stimuli. This study generally supports the hypothesis that voice quality is an important enhancement cue for pitch range.

## Introduction

Pitch perception is crucial to speech processing, as speakers use pitch to communicate important linguistic information like tone and intonation. Although pitch refers to an auditory property, in speech studies the term is often used interchangeably with its acoustic correlate, fundamental frequency (F0). At the same time, speakers differ in F0 ranges such that there may be overlap in the acoustic signals of “high” and “low” F0 for different speakers, as well as for different speakers’ phonetic (e.g., tonal) categories. In order to ascertain the linguistic pitch intended by a speaker, listeners must effectively locate the pitch within its speaker’s pitch range.

Speaker normalization has been known as a challenge for automatic tone recognition by a machine, yet it is an effortless process by human listeners. Speaker normalization is certainly easier when listeners are previously exposed to a voice or when the context is available (e.g., Wong and Diehl, 2003). However, studies (e.g., Honorof and Whalen, 2005; Lee, 2009; Lee et al., 2010) have shown that speaker normalization is even more efficient and effective than previously assumed, as listeners are able to identify the pitch location of very brief voice samples (e.g., only six glottal periods available) in an unknown speaker’s range, without any contextual cues. This suggests that listeners must use other signal-internal information that co-varies with F0 as cues to perceive pitch range.

Both Honorof and Whalen (2005) and Lee et al. (2010) speculated that voice quality, defined as the variability in the spectrum due to the variability of glottal constriction and vocal-fold contacts, could be such a cue. This speculation is plausible because systematic co-variation between F0 and voice quality has been found in both speech production studies (e.g., Kuang, 2017) and singing studies (e.g., Hollien and Michel, 1968; Hollien, 1974; Titze, 1988; Roubeau et al., 2009). That is, voice quality continuously changes as a speaker’s F0 increases or decreases in a nonlinear but predicable manner, and certain pitch ranges are bound to certain types of voice quality. For example, the lowest pitch range is often associated with vocal fry, and the highest pitch range is associated with tense voice and falsetto.

Indeed a study based on Mandarin speakers (Lee, 2009) found that voice-quality-related spectral cues (i.e., H1-H2, the relative amplitude difference between the first harmonic and the second harmonic; and H1-A3, the amplitude difference between the first harmonic and the third formant) were correlated with tone classification between high and low. However, they further noted that F0 was the only significant predictor for identification accuracy in the regression model. Bishop and Keating (2012) replicated Honorof and Whalen’s (2005) experiment and found that acoustic measures of voice quality had only a very small effect on pitch location ratings. They suggested that voice quality only indirectly influences pitch perception, possibly through its information about sex. This is plausible, since talker processing has been shown to interact with linguistic processing (e.g., Mullennix and Pisoni, 1990). However, since a multi-speaker design was used in these previous studies, and voice quality cues were not explicitly manipulated and controlled, it is impossible to tease apart its indirect gender effect (i.e., through the additional processing of the talker’s gender) from its direct signal-internal effect (i.e., through the co-variation between pitch and voice quality). Therefore, although the co-variation between pitch and voice quality has been found in production studies, it remains to be shown whether such co-variation relationship also exists in speech pitch perception.

Nonetheless, outside of speech studies, psychoacoustic studies have generally suggested that spectral properties (usually referred as “timbre” in this body of literature) of the signal can directly interfere with the perception of pitch height (e.g., Melara and Marks, 1990; Krumhansl and Iverson, 1992; Singh and Hirsh, 1992; Allen and Oxenham, 2014; to cite a few). A common finding from these studies is that there are interactions between pitch and timbre in speeded classification tasks. Listeners were instructed to attend to either timbre changes or pitch changes, while both dimensions simultaneously varied. Listeners’ pitch classification was more accurate and faster when the timbre dimension was “congruent” with the F0 dimension. Various types of spectrum have been explored in this body of literature, and have been found to be able to interfere with pitch perception: for example, natural timbres from different musical instruments (e.g., Krumhansl and Iverson, 1992; Marozeau et al., 2003); different values of duty cycles of square waves (Melara and Marks, 1990); the location of the center frequency of harmonic complex tones (e.g., Warrier and Zatorre, 2002; Russo and Thompson, 2005; Silbert et al., 2009; Allen and Oxenham, 2014); and the spectral locus of complex tones (Singh and Hirsh, 1992). Although various types of timbre have been tested, most studies only used non-speech stimuli, while speech-related studies are relatively rare. Therefore, it remains unclear whether spectral information is integrated in speech-related pitch perception as well, and it is possible that listeners ignore spectral cues in speech tasks, as speech is subject to very different neural processing. For example, studies have shown that listeners behave differently when processing speech and non-speech stimuli (e.g., Liberman, 1970; Repp, 1982), and neural imaging studies have, similarly, found that people use different parts of the brain to process linguistic and non-linguistic pitch (e.g., Merrill et al., 2012).

Although speech-related studies on the interaction between spectrum/timbre and pitch are very rare, Stoll (1984) and Krishnan et al. (2011) showed that timbre and pitch are probably integrated in the speech domain as well, since pitch perception is influenced by the manipulation of vowel formants, which is known to influence the overall shape of the spectrum. It is worth pointing out that there is a co-variation between vowel height and F0 in production as well; high vowels are naturally produced with higher F0 (e.g., Whalen and Levitt, 1995). Linguistically meaningful spectral variation is not only limited to vowel quality, as other dimensions such as voice quality also significantly affect the shape of the vocal spectrum. Therefore, it remains to be shown what kind of linguistically meaningful spectral variation is integrated into the perception of linguistic pitch targets. Specifically, in this study, we ask whether voice quality can function as an indicator of pitch range and therefore affect the perception of pitch height.

Taken together, in linguistic studies, it remains unclear whether and how voice quality cues interfere with linguistically meaningful pitch perception (e.g., tone perception); in psychoacoustic studies, it remains unclear whether the interaction between timbre and pitch occurs in the domain of speech as well, and if so, whether speech mode plays a role. The present study bridges the gaps in the linguistic and psychoacoustic literature in those respects.

The voice quality cue that was tested in this study is spectral slope. It has been well established that the relative slope of the voice source spectrum is one of the most important acoustic correlates of voice quality (see Gobl and Ni Chasaide, 2012 for a general review). A relatively steep spectral slope is associated with a breathier voice, and a flat spectral slope is associated with a tenser or creakier voice (the latter also characterized by pulse-to-pulse variability). The spectral tilt is usually measured as the amplitude of the fundamental (H1) relative to some higher-frequency components (e.g. H1-H2, H1-A1, H1-A2, and H1-A3; A1, A2, A3 are the amplitudes of the harmonic near the first, second and third formants). These measures have been found to be the reliable indicators of phonation contrasts across languages (e.g. Southern Yi: Kuang and Keating, 2014; Green Mong: Andruski and Ratliff, 2000; White Hmong: Esposito, 2012; Takhian Thong Chong: DiCanio, 2009; Sui/Kuai: Abramson et al., 2004; Javanese: Thurgood, 2004; Ju| ’hoansi: Miller, 2007; Santa Ana Valle Zapotec: Esposito, 2010; Mazatec: Garellek and Keating, 2011; Gujarati: Khan, 2012), and of voice quality classification in perceptual spaces (e.g., Kreiman et al., 2007, 2014; Garellek et al., 2016). Therefore, the working hypothesis of the current study is that, if voice quality can affect pitch perception, manipulating the spectral slope of a voice should be able to shift listeners’ perception of pitch height. This hypothesis is tested with both non-speech and speech stimuli.

The stimuli in this study were designed to resemble the prosody of natural utterances. The F0 contours (c.f. Method section for details) which contains two F0 peaks are similar to the design in previous studies on prominence perception (e.g., Terken, 1991; Gussenhoven et al., 1997). One question raised in those studies was that how listeners perceived the relative prominence of the two F0 peaks, whether they relied more on the local pitch targets (such as comparing with the other peak), or more on the global pitch range (the overall pitch height of the utterance within the speaker’s range). It was found that both global and local target play important roles in prominence perception (Gussenhoven et al., 1997). Although our study does not explicitly refer to prominence, a similar question can be also examined here, if voice quality does contribute to the pitch height normalization, whether it contributes to the normalization of the global pitch range or the normalization of the local pitch targets; and furthermore, whether speech mode plays any role in the normalization strategies.

## Experiment 1: Pitch Perception With Non-Speech Stimuli

### Materials and Methods

#### Stimuli

**FIGURE 1:**
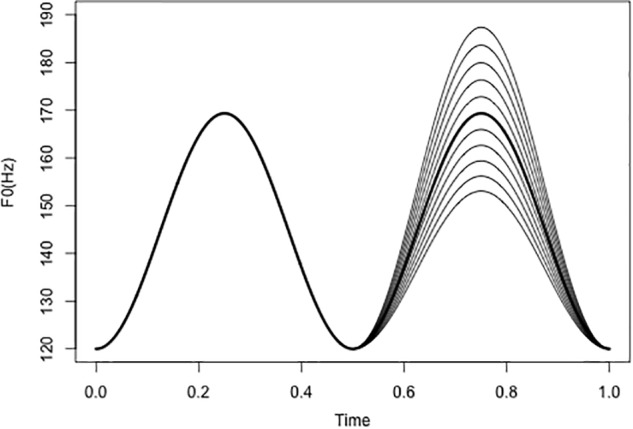
Manipulation of F0 contours with two peaks. The minimum F0 of the contours is 120 Hz; the first peak has a constant F0 value at 169.34 Hz, and the second peak is a continuum with 11 steps (0.35 semitone/step). Peaks 1 and 2 are identical at step 6 (dark line in the second peak).

Similar to our previous pilot study (Kuang and Liberman, 2015), complex tones varying in pitch and spectral cues were synthesized. The stimuli were four sets of sine-wave overtones with two peaks, which were created by convolving a hamming window with a sawtooth whose baseline F0 value is always 120 Hz. The pitch contour was designed to simulate the prosody of natural utterances. To manipulate the F0 cues, the F0 of the first peak is always set to 169.34 Hz, while the second peak is a pitch continuum with 11 steps between 153.06 and 187.36 Hz, with an interval of 0.35 semitones. At step 6, peak 1 and peak 2 are identical in F0. The F0 range of these pitch contours roughly covers the upper half of the comfortable pitch range of a male speaker (Baken and Orlikoff, 2000). Pitch manipulation is illustrated in Figure 1.

**FIGURE 2:**
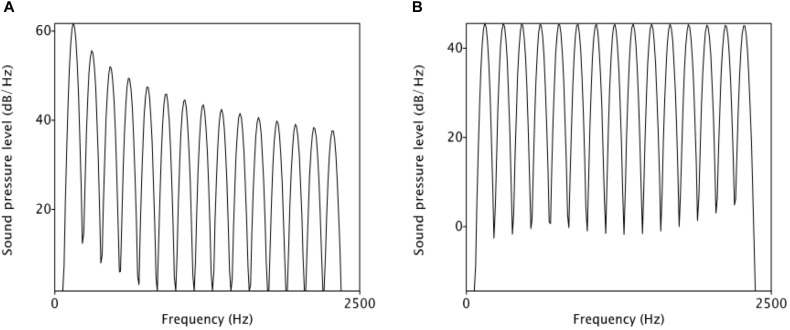
Spectrum manipulation: tilted spectrum **(A)** vs. flat spectrum **(B)**.

To manipulate voice quality-related spectral cues, two source spectra, one with tilted slope and the other one with flat slope, were first created. In the tilted spectrum, overtone amplitude decreases with an 1/F slope, to a point 15 dB below the fundamental (Figure 2A). As can be seen here, as a result of the tilted slope, the first harmonic is relatively more prominent than the higher-frequency harmonics. By contrast, in the flat spectrum (Figure 2B), the overtone amplitude is kept constant, so the first harmonic is not prominent in the spectrum. Using the voice quality terms, the flat spectrum, which has more energy in high-frequency harmonics than the tilted spectrum, indicates a tenser voice.

The two types of source spectrum were then applied to the two peaks of the complex tones and resulted in four spectral conditions, as summarized in Table 1. Intended voice quality combinations were indicated in relative terms.

**Table 1:** Summary of manipulations of the stimuli.

Set	Peak1 spectrum	Peak2 spectrum	Intended VQ combination
Set BB	Tilted	Tilted	Breathier + Breathier
Set TT	Flat	Flat	Tenser +Tenser
Set BT	Tilted	Flat	Breathier + Tenser
Set TB	Flat	Tilted	Tenser + Breathier

*Tilted spectrum, original spectrum; flat spectrum, boosted spectrum.*

Therefore, there were 44 distinct stimuli (11 F0 steps × 4 spectral conditions) in total. All stimuli were 1 s in duration.

#### Procedure

A forced-choice pitch classification task was used to test how listeners categorize pitch values in different spectral conditions. Ten copies of each stimulus were presented in random order to each listener. For each trial, the listeners were asked to attend to pitch, and judge whether the second peak is higher or lower than the first peak by clicking on the corresponding buttons on the computer screen. All testing took place in a soundproof booth with stimuli presented over Sennheiser 280 headphones.

#### Subjects

Fifty eight participants, aged between 18 and 22 (half females), were recruited from the student population at the University of Pennsylavnia. All of them reported to speak English as their primary language. None of them reported to receive extensive musical training. Three of them failed to complete the task as instructed (i.e., clicked on the same answer for all trials), and thus were excluded from the analysis. None of the participants reported to have hearing issues.

### Predictions

**FIGURE 3:**
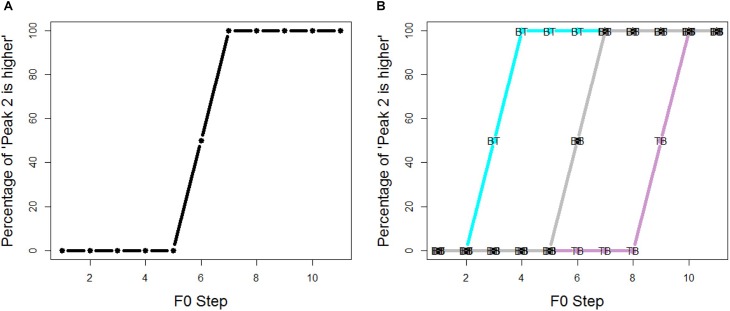
Predictions of the experiments: **(A)** if listeners do not pay attention to spectral cues, there is no shift in the pitch classification function; **(B)** if listeners do pay attention to spectral cues, there should be a signficant shift in the pitch clasification: set BT gets the most “peak 2 is higher” responses, while set TB gets the fewest “peak 2 is higher” responses.

Figure 3 depicts the predictions of the experiment; as shown in Figure 3A, if listeners do not pay attention to spectral cues, there is no shift in the pitch classification function. On the other hand, if listeners indeed pay attention to spectral cues, there should be a signficant shift in the pitch clasification, as indicated in Figure 3B. Set BT (tiled/breathier + flat/tenser) would receive the most “peak 2 is higher” responses, while set TB would motivate the fewest “peak 2 is higher” responses. Note that, despite the way Figure 3 is plotted, we do not assume a categorical perception of the pitch classifiction.

### Results

**FIGURE 4:**
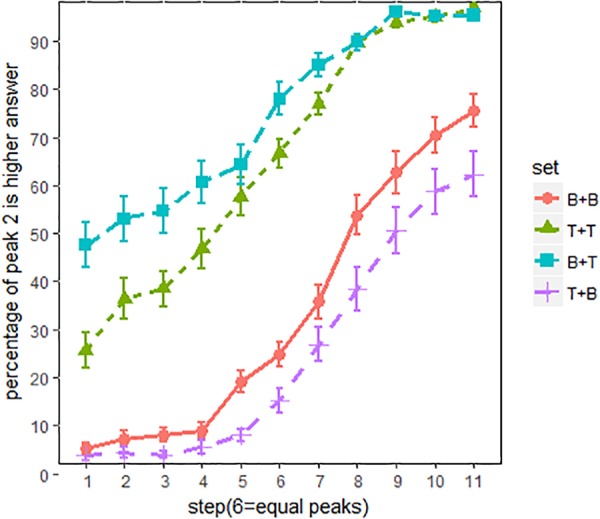
Pitch classification functions for all listeners. x-axis = F0 steps, y-axis = proportion of “peak 2 is higher” responses; line patterns are different spectral conditions. The error bars are 95% confidence intervals.

Figure 4 shows the proportion of “peak 2 is higher” responses across all listeners. The main effects of spectral conditions were evaluated using an MCMC generalized linear mixed-effects model (*mcmcglmm* package in R). F0 steps (1–11) and spectral conditions (BT, BB, TT and TB) were the fixed factors, and random intercepts and slope were included for subjects. Main effects of spectral conditions were summarized in Table 2. The results were reported as means of regression coefficients, followed by 95% highest posterior density intervals in square brackets and associated *p*-values. As shown in Table 2, significant effects were found between every two spectral conditions, which means that pitch classification function is significantly shifted in each spectral condition. The proportion of “peak 2 is higher” responses was in the order of Set BT (tiled/breathier + flat/tenser) > Set TT (flat/tenser + flat/tenser) > Set BB (tilted/breathier + tilted/breathier) > Set TB (flat/tenser + tilted/breathier; see Figure 3).

**Table 2:** Main effects of spectral conditions between every two conditions.

	BB	TT	BT
TT	1.3[1.2,1.5], *p* < 0.001		
BT	1.7[1.6,1.8], *p* < 0.001	0.4[0.3,0.6], *p* < 0.001	
TB	0.4[0.3,0.5], *p* < 0.001	1.8[1.7,2.0], *p* < 0.001	2.5[2.4,2.7], *p* < 0.001

*Means of regression coefficients, followed by 95% highest posterior density intervals in square brackets and associated p-values*.

Overall, the perception of pitch height was strongly biased by the spectral cues. As can be seen in Figure 4, compared to set BB, pitch classification function for set BT (breathier + tenser) was dominated by the “peak 2 is higher” responses, even when peak 2 was about 10 Hz lower than peak 1. By contrast, pitch classification function of set TB (tenser + breathier) was shifted in the opposite direction. In this condition, listeners hardly heard a higher peak 2, even when peak 2 was about 10 Hz higher than peak 1. In other words, when the second peak was tenser than the first peak, listeners tended to perceive a higher pitch, and when the second peak was breathier than the first peak, they tended to perceive a lower pitch. Interestingly, pitch classification functions for set BB (breathier + breathier) and set TT (tenser + tenser) were also significantly different, with set TT more in favor of “peak 2 is higher”. This suggests that listeners were also sensitive to the overall “voice quality” of the utterances.

## Experiment 2: Pitch Perception With Speech Stimuli

### Materials and Methods

#### Stimuli

**FIGURE 5:**
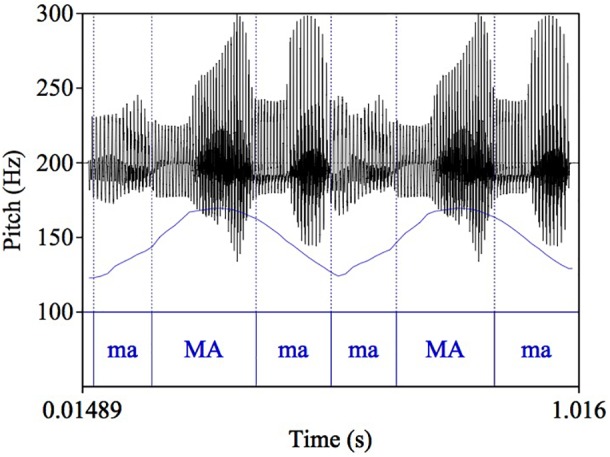
Example of speech stimuli: a phrase with two “maMama” words.

The basic design of the stimuli is comparable to the first experiment – four sets of utterances with two F0 peaks differing in spectral conditions. As shown in Figure 5, in the second experiment, each F0 peak was carried by three /ma/ syllables, with a stressed syllable in the middle aligned with the highest F0, so that the whole six-syllable sequence had the prosodic pattern of a phrase like “phonetic condition” or “electric banana,” which have a natural LHL-LHL pitch pattern.

The stimuli were resynthesized from the natural production of a male English speaker. The speaker was asked to produce a pseudo-word /ma.’ma.ma/ with the same intonation pattern as “banana,” which naturally has a LHL F0 contour (i.e., single peak). The two-peak “maMAma maMAma” phrase was resynthesized from the single peak token, so that all the segmental features are identical between the two “maMAma” words.

**FIGURE 6:**
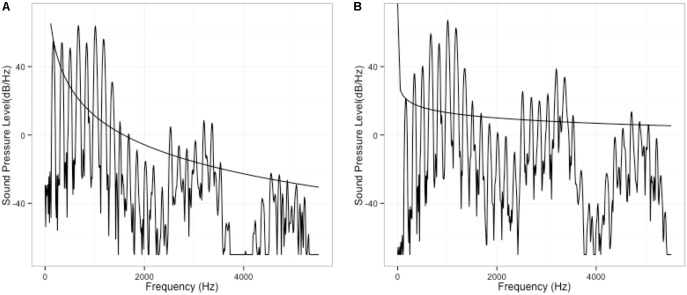
Spectral manipulation: **(A)** original; **(B)** boosted. The spectral slope of **(B)** is 6 dB/octave greater than that of **(A)**.

In order to preserve the naturalness of the original utterance, the TANDEM-STRAIGHT algorithm (Kawahara et al., 2008) was used for resynthesis. With the algorithm, the interference with periodicity is minimized while smooth spectral envelope can be extracted for resynthesis. Before the manipulation, a single token of /ma.’ma.ma/ was first analyzed into F0 component and spectral component, and two components were then manipulated independently. F0 manipulation is the same as the first experiment, as illustrated in Figure 1. The first F0 peak (i.e., first maMAma) of the phrase was kept constant, and the second peak consists of 11-step F0 continuum. To manipulate voice quality cues, two versions of spectral slope were created for the single “maMAma” token: one version with more high-frequency energy, and thus a flatter spectrum (i.e., tenser voice), and the other version with less high-frequency energy, and thus a more tilted spectral slope (i.e., breathier voice), comparable to the first experiment. The breathier version was the original spectrum of the natural production, while the tenser version was modified such that the slope of the spectrum was 6 dB/octave greater than the original slope. The result of this spectral boost is depicted in Figure 6.

The two types of source spectrum were again applied to the two-peak phrase and resulted in four spectral conditions, same set-up as in Table 1. All stimuli were 1s in duration. In a word, the stimuli in experiment 2 were the same as experiment 1, except that the stimuli are speech.

#### Procedure

Same as the first experiment, a forced-choice pitch classification task was used to test listeners’ categorization of pitch values under different spectral conditions. Five copies of each stimulus were presented in random order to each listener. For each trial, the listeners were asked to focus on pitch and to evaluate whether the second “maMAma” word was higher or lower than the first one by clicking on the corresponding buttons on the computer screen. To introduce the idea of linguistically meaningful pitch to an English speaker, we used examples from English intonation. For example, the phrase “my name” is higher in “Anna may know my name?” than in “Anna may know my name.” These two sentences have identical pitch accents and other prosodic aspects, except for pitch contour. The participants were asked to produce the example sentences themselves and to judge which “my name” was higher. In the following practice session, examples from set BB, in which both F0 peaks have the same spectral property, were used to demonstrate the task. These procedures encouraged listeners to attend to pitch difference but not to other cues, such as intensity. The experiment was run with Qualtrics’ online survey system. The subjects were instructed to conduct the procedure with headphones or earbuds. This online tool was utilized for the convenience of recruiting participants, as this study is part of a larger cross-linguistic study. Moreover, the results of Experiment 1 were faithfully replicated through Qualtrics in our previous pilot study (Kuang and Liberman, 2015), we therefore believe the results collected from the online tool are valid.

#### Subjects

Another 34 English speakers between age 18 and 22 (half females) were recruited from the student population at the University of Pennsylvania. All the subjects reported to have normal hearing and speaking. None of the listeners in this study reported to have professional musical (either vocal or instrumental) training.

### Results

Figure 8 shows the proportion of “peak 2 is higher” responses for all English listeners. The primary effects of spectral conditions were evaluated using an MCMC generalized linear mixed-effects model. F0 steps (1–11) and spectral conditions (BT, BB, TT, and TB) were used as fixed factors, and random intercepts and slopes were included as subjects. The main effects of the spectral conditions are summarized in Table 3. As shown in Table 3, pitch classification functions shifted significantly in sets BT and TB, relative to BB and TT.

**Table 3:** Main effects of spectral conditions for every pair of conditions.

	BB	TT	BT
TT	0.17[-0.05,0.44], *p* = 0.17		
BT	1.1[0.5,1.9], *p* < 0.001	1.03[0.7,1.4], *p* < 0.001	
TB	0.5[0.3,0.7], *p* < 0.001	0.4[-0.7, -0.1], *p* < 0.001	1.5[-2.1, -0.9], *p* < 0.001

*Means of regression coefficients followed by 95% highest posterior density intervals in square brackets and associated p-values*.

**FIGURE 7:**
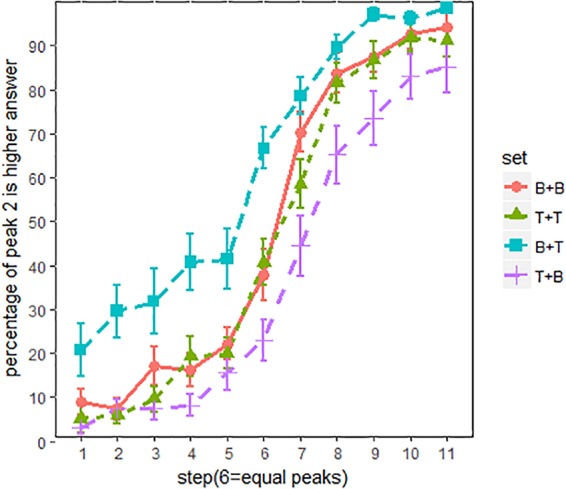
Pitch classification functions for English listeners. x-axis = F0 steps, y-axis = proportion of “peak 2 is higher” responses; line patterns denote different spectral conditions. Error bars denote 95% confidence intervals.

Overall, the current experiment succeeded in replicating the previous experiment’s results (Figure 3), as spectral cues exerted strong influence on pitch height perception. Compared with set BB and TT (Figure 7), in which the two peaks are identical with respect to spectral conditions, the pitch classification function for set BT (breathier + tenser) was composed largely of “peak 2 is higher” responses. Contrastively, the pitch classification function of set TB (tenser + breathier) was opposite. When the second peak was tenser than the first, it was generally perceived as being higher in pitch; when the second peak was breathier, it was generally perceived as lower in pitch.

However, there is also a noticeable difference between Figures 3, 6. When using non-speech stimuli (Figure 3), set TT also diverged from set BB. This suggests that the second peak’s spectral condition alone exerts a strong effect on pitch perception. However, when using speech stimuli (Figure 7), set TT and set BB no longer strongly differ from one another. This suggests that listeners were inattentive to the absolute quality of the utterance but paid more attention to the relative difference between the two peaks. Additionally, although sets BT and TB both significantly shifted, set BT (breathier + tenser) had a greater effect than set TB (tenser + breathier). This suggests a perceptual bias whereby a tenser second peak results in a stronger effect.

Because listeners appeared to be highly sensitive to spectral differences, the question remains whether a shift will still occur if the spectral difference between the two peaks is diminished. To investigate, a third experiment was conducted in which the spectral differences in Experiment 2 were halved.

## Experiment 3: Replicate Experiment 2 With Smaller Spectral Difference

The design and procedure in Experiment 3 were exactly the same as in Experiment 2, except that the spectral difference was only 3 dB/octave, half of the 6 dB/octave difference used in Experiment 2. An additional 30 listeners (half females) were recruited from the student population to participate in the experiment.

**FIGURE 8:**
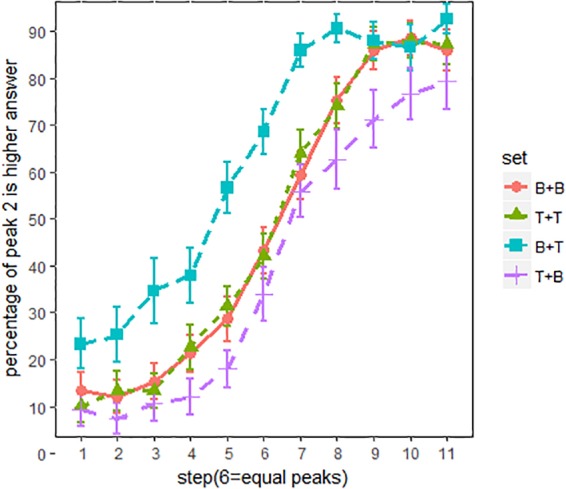
Pitch classification functions for English listeners. Spectral difference is 3 dB/octave. x-axis = F0 steps, y-axis = proportion of “peak 2 is higher” responses; line patterns denote different spectral conditions. Error bars denote 95% confidence intervals.

Table 4 summarizes the main effects of the spectral conditions. Similar to Table 3, sets TB and BT significantly shifted from sets A and B. This effect can be seen clearly in Figure 8. Although the spectral difference was much smaller in experiment 3, the salience of the effect remained, as shown in Figure 8. This suggests a high degree of listener sensitivity to spectral difference.

**Table 4:** Main effects of spectral conditions for every pair of conditions, after reducing the spectral difference to 3 dB/octave.

	BB	TT	BT
TT	0.01 [0.18,0.22], *p* = 0.9		
BT	0.7[0.5,0.9], *p* < 0.001	1.7[0.9,2.5], *p* < 0.001	
TB	0.45[0.24,0.67], *p* < 0.001	1[0.5,1.7], *p* < 0.001	1.4[1.0,1.8], *p* < 0.001

*Means of regression coefficients followed by 95% highest posterior density intervals in square brackets and associated p-values*.

**FIGURE 9:**
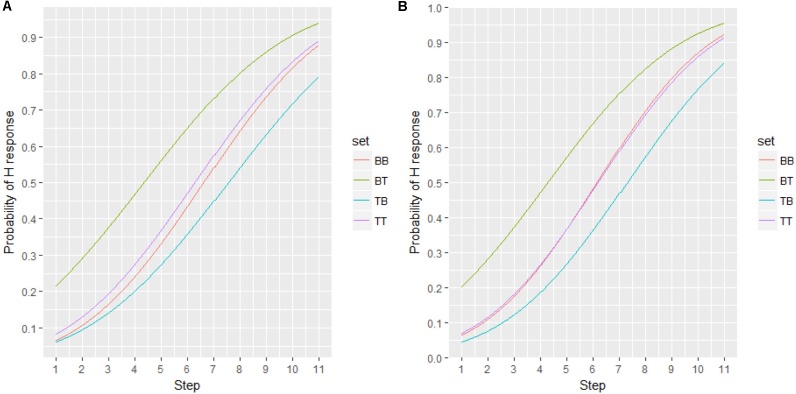
Fitted curve for experiment 1 **(A)** and 2 **(B)**.

To measure the degree of shift, the classification functions were fitted with a sigmoid function in order to determine the threshold (alpha, i.e., left-to-right shift) and slope (beta) of the response probability. Figure 9 displays the curves fitted to the response probabilities of Experiments 2 and 3, with threshold and slope values displayed in Table 5.

**Table 5:** Threshold (α) and slope (β) of the fitted sigmoid function.

Set	Exp1	Exp2
	**α**	**β**	**α**	**β**
BB	6.4	2.0	6.3	1.9
TT	6.6	2.0	6.2	1.8
BT	4.4	2.5	4.3	2.1
TB	7.6	2.2	7.2	2.0

As suggested by Table 5, Experiments 2 and 3 have similar results despite the reduced spectral differences between the two peaks in Experiment 3. In both experiments, set BT shifted to steps to the left from TT and BB (i.e., alpha.set BT – alpha.set BB/TT = -2), suggesting that altering the spectral condition resulted in listeners perceiving the stimuli as 0.7 semitones higher (0.35 semitones/step × 2). Conversely, set TB shifted one step to the right of BB and TT (alpha.set TB – alpha.set BB/TT = 1), indicating that these stimuli were perceived as 0.35 semitones lower by speakers.

## General Discussion

This study’s major contribution is demonstrating that altering spectral slope significantly affects pitch height perception. It therefore strongly supports the hypothesis that voice quality cues substantially contribute to relative (peak) pitch perception. Listeners tend to perceive a higher relative pitch for a peak whose high-frequency components have more energy (indicating tenser voice quality) than for a peak of identical F0 but with less high-frequency energy. The direction of the shift is consistent with the naturally co-varying relationship between F0 and voice quality, whereby tense voice produces a higher F0 (e.g., Kuang, 2017).

It should be noted that non-speech psychoacoustic studies (e.g., Allen and Oxenham, 2014) have also found that the location of the spectral centroid can affect listeners’ pitch height perception. Specifically, a low frequency emphasis in the spectrum leads to “duller” and lower sound, while higher frequency emphasis in the spectrum leads to a “brighter” and higher sound. Although the shape of spectrum in previous studies differs from our current study, and not related to speech, the direction of the perceptual shift is fairly consistent with our study. One question worth asking is why there is such a correlation between the spectrum with more high-frequency energy and high pitch. In light of the co-variation between voice quality and pitch found in both production (e.g., Titze, 1988; Kuang, 2017) and perception, we speculate that the correlation between pitch and spectrum probably evolves from the interaction between speech production and perception. That is, the auditory system has tuned to adapt the co-varying cues in pitch production, as human auditory system is evolved to be especially sensitive to speech-related signal (e.g., Lieberman, 1991).

Furthermore, speech mode does have an effect on how the spectral cues are integrated in pitch perception. Overall, listeners employ a rather global strategy for the non-speech stimuli, while utilize a local strategy for the speech stimuli. In the first experiment, in which non-speech stimuli were used, set BB and set TT (the controlled conditions where the two F0 peaks have identical spectrum) shift away from each other significantly, with set TT (tenser + tenser) prompting a greater number of “peak 2 is higher” responses. This suggests that the absolute spectral condition of the entire utterance has a strong effect on pitch perception. However, as shown in Experiments 2 and 3, set BB and set TT do not differ from each other, which suggests that when listeners are in speech mode, they are less sensitive to the absolute quality of the entire stimulus, and are instead more sensitive to the relative differences within the stimulus. This suggests that the absolute difference between set TT and BB have been normalized by listeners. This normalization strategy is helpful for speech processing because overall voice quality variation among speakers is not linguistically meaningful.

In addition, in Experiment 3, the robustness of the spectral cue was evaluated by reducing the spectral difference between the two peaks’ F0. The results indicate that even a small spectral difference can result in a significant shift in the pitch classification function, suggesting that listeners are highly attentive to voice quality cues. It should be noted that in this experiment, surrounding F0 contours for the pitch targets were given, so the pressure of using voice quality cues is much lesser than in previous pitch-location experiments (e.g., Honorof and Whalen, 2005; Lee, 2009), in which only very brief sound samples without any context were presented. Yet, listeners’ pitch perception was significantly affected by the manipulation of spectral slope.

Finally, it is notable that the shift of set BT (breathier + tenser) is greater than set TB (tenser + breathier) for Experiments 2 and 3, which points to a perceptual bias – when the second peak is tenser, people are more likely to hear the second peak being higher. This perceptual bias is comparable to the perceptual bias based on F0. In many languages such as English and Mandarin (e.g., Ladd, 1984), there is a global trend of F0 contours drifting downwards over the course of an utterance arguably due to a drop in the subglottal pressure (e.g., Lieberman, 1966). As demonstrated by Pierrehumbert (1979), F0 declination is expected and used in pitch perception by English listeners. In a pitch classification task, listeners were asked to judge whether the second peak of a two-peak f0 contour is higher or lower than the first peak. When listeners judged that the two peaks had identical pitch, the second peak actually had a lower F0. The stronger effect for the breathier + tenser set found in the current study suggested that, just as English speakers expect F0 declination, they probably also expect a declination in tenseness.

The findings of this study have important implications for speech prosody studies. Voice quality plays a crucial role in prosodic structure, as it is a part of pitch processing. Pitch can no longer be construed as synonymous with F0, either in speech production or in perception, since linguistic pitch perception is in fact generally determined by both F0 and voice quality cues. Thus, what is perceptually “higher” does not necessarily have a higher F0 in the signal. For example, in English, tense voice is related to the production of high-pitched and prominent positions, such as lexical stress and high-type pitch accents (e.g., Pierrehumbert and Talkin, 1992; Epstein, 2002; Ni Chasaide et al., 2013; Garellek, 2014). Based on the findings from this current study, it is reasonable to speculate that tense voice also plays a role in the perception of pitch accents and stress.

Moreover, because of the co-variation between voice quality and F0 (Kuang, 2017), voice quality can provide useful information about the relative pitch location within a speaker’s pitch range. Globally speaking, a tense voice basically indicates that the speaker is speaking in his/her high range, while vocal fry indicates that the speaker is speaking in his/her low range (Kuang and Liberman, 2016); and more locally, a relatively tenser voice can indicate a relatively higher pitch. Therefore, voice quality cues can function as an enhancement cue in pitch perception. so that intended tonal targets (especially extreme pitch targets such as extra high and low) are more easily perceived. This claim is consistent with the findings from tone perception studies from various languages. For example, it has been shown that the presence of voice quality cues (e.g., allophonic vocal fry or tense voice) can facilitate low tone perception (e.g., Cantonese: Yu and Lam, 2014; Mandarin: Yang, 2011; Black Miao: Kuang, 2013) and extra high tone perception (e.g., Black Miao: Kuang, 2013), all in a multi-speaker setting.

In conclusion, this study demonstrates that voice quality is an important part of pitch perception, and that listeners actively take advantage of these cues in their production and perception of prosodic structures, as they are useful in resolving intended linguistic pitch targets in speech. To take the larger picture into consideration, this study provides a better understanding of the interaction between F0 and voice quality, and sheds light on a more fundamental question of why voice quality is useful to prosodic structures, and when they are likely to occur. Of course, there are some limitations with this study and more factors will be taken into account in our future studies. For example, spectral cues are only one acoustic aspect of voice quality, and therefore future studies should include other acoustic cues of voice quality, such as noise and periodicity. Noise is important for the perception of breathy voice, and periodicity is important for the perception of creaky voice. Finally, future studies should screen for listeners’ musicality, as listeners’ sensitivity to F0 can have significant effects on pitch perception.

## Ethics Statement

The protocol was approved by the IRB board at University of Pennsylvania. All subjects gave written informed consent in accordance with the Declaration of Helsinki.

## Author Contributions

JK contributed to the conception and the design of the study. JK and ML designed and created the stimuli. JK conducted the experiments, analyzed the data, and wrote the paper. Both authors read and approved the submitted manuscript.

## Conflict of Interest Statement

The authors declare that the research was conducted in the absence of any commercial or financial relationships that could be construed as a potential conflict of interest.
